# Students’ perceptions towards self-directed learning in Ethiopian medical schools with new innovative curriculum: a mixed-method study

**DOI:** 10.1186/s12909-019-1924-0

**Published:** 2020-01-08

**Authors:** Haftom Hadush Kidane, Herma Roebertsen, Cees P. M. van der Vleuten

**Affiliations:** 1 Department of biomedical science, Aksum University, Aksum, Ethiopia; 2 Department of Educational Development and Research, Faculty of Health, Medicine and Life Sciences, Maastricht University, Maastricht, The Netherlands

**Keywords:** Self-directed learning, Preclinical students, Hybrid curriculum, Ethiopia

## Abstract

**Background:**

Self-directed learning (SDL) is an appropriate and preferred learning process to prepare students for lifelong learning in their professions and make them stay up-to-date. The purpose of this study was to explore preclinical students following a hybrid curriculum in Ethiopia experiences to SDL and the support of several learning activities from the curriculum on their SDL. A mixed-method research design was employed.

**Methods:**

Quantitative data were collected by using a self-administered questionnaire of 80 items measuring students’ perceptions on their SDL capability as well as to explore students’ views about the influence of components of the curriculum on their SDL. Additional two focus group discussions, each containing eight participants from year-1 and year^− 2^ students, were conducted. The quantitative data were analyzed using SPSS. The focus group discussions were reviewed, coded, and then thematically analyzed.

**Results:**

Our study showed a significant increase in SDL score on comparing students at year-1 with students at year-2 (*p = 0.002*). Both year-1 and 2 students rated PBL tutorial discussion and tutors had high influence on their individual learning; whereas, other curricular components such as lectures and testes had low influence on their SDL ability. PBL tutorial discussion and module objectives showed strong correlation with students’ SDL scores, r = 0.718 & r = 0.648 (*p* < 0.01), respectively. Besides, PBL tutorial discussion was found strongly correlated with tutors (r = 0.599 (*p* < 0.01)) and module objectives (r = 0.574 (*p* < 0.01)). Assessment was highly correlated with lectures (r = 0.595 (*p* < 0.01)). Findings from qualitative data showed that certain curricular components played role in promoting students’ SDL. Tutorials analyzing problems played a major role on students’ self-directed learning abilities.

**Conclusions:**

Although the study implied that components of the hybrid curriculum, mainly PBL, could encourage preclinical students’ self-directed learning, the curriculum is still not free from teacher-centred culture as the majority of teachers still have high power in deciding the learning process. A further longitudinal study is needed to verify the actual level and ability of medical students’ SDL.

## Background

Equipping medical professionals with skills essential for lifelong learning is immensely required [1]. In today’s society, as scientific knowledge constantly develops, medical professionals should be able to learn and upgrade their competency from time to time [2]. Self-directed learners have an adequate amount of accountability to select the content to be studied, to assign time for it, and to understand the content deeply using any method both inside and outside class [3]. Students mostly need training and support to become a self-directed learner [4, 5]. This support is necessary when students are teacher dependent and not ready at the beginning for self-directed learning (SDL) until they pass through a series of progressive stages [4].

To help learners persistently improve SDL skills, implementation of appropriate teaching strategies and activities has paramount importance [4]. Several studies have indicated different teaching strategies and activities that motivate students toward self-directed learning [6–8]. Problem-based learning (PBL) is one of such widely accepted educational strategies that stimulate SDL [8]. Evidence exists that PBL promotes SDL because learners are responsible to plan, monitor, and evaluate their learning process [9–11]. Within a PBL strategy, students usually work together in tutorials analyzing real-world problems [12]. These tutorial authentic problems promote students’ independent responsibility for learning and help connect them with the large world beyond the classroom [12, 13].

To become a self-directed learner, there are promoting and deterring conditions related to the type of offered curriculum, and students’ and teachers’ level of understanding about SDL [14, 15]. Unlike innovative curricula, a conventional curriculum is not likely to produce self-directed learners [16]. A large body of research indicated graduates of PBL curricula are better self-directed learners [17, 18]. On the other hand, there are inconsistent reports in different settings about the effect of a hybrid PBL curriculum on students’ SDL ability [19]. By hybrid curriculum is meant a curriculum that combines traditional teaching methods and PBL aiming to benefit from advantages of the innovative learning strategy [8, 20]. Besides curriculum approach, there is no common consensus between the correlation of sociodemographic factors such as curriculum year, age, and academic achievement and students’ SDL [21–28]. Leatemia et al. [25] revealed higher level of SDL readiness in first-year students compared to second-year medical students of a hybrid PBL curriculum. Novertheless, Frambach et al. [23] noted that students from three medical schools (two had PBL-curriculum and one had hybrid PBL curriculum) in different cultures progressively accustomed the principle of SDL from year to year. Premkumar and colleagues [28] indicated a significant effect of age on SDL ability: higher age medical students were more self-directed learners compared with students from the same cohort of younger age. On the contrary, other studies have revealed no correlation between student age and SDL skill [25, 27]. The relation between academic performance and SDL is still not clear; some studies reported strong correlation [22, 24, 26] while others reported weak relation [21].

The feasibility of SDL in all cultural learning environments has also been an area of discussion. Culture influences students’ self-direction in learning [23, 29–33]. Thus, studying the effect of a hybrid curriculum on adult learners’ SDL in the African setting is necessary.

So far, there are no studies conducted which investigate the effectiveness or level of self-directed learning in Ethiopian students in a hybrid curriculum like the New Innovative Medical Curriculum (NIMC) schools. The purpose of this study is, therefore, to explore how adult preclinical students’ in Ethiopian culture experienced SDL and to see if they experienced the influence of the various elements of the hybrid curriculum on their SDL skill. We assumed that culture would influence SDL and year-two students to have higher levels of SDL ability than year-one students. Because year-one students are not ready for complete self-direction compared to students in year-two who are relatively with high experiences in PBL and supposed to pass through a series of progressive stages. Besides, we supposed that the effect of age and academic performance between students in year-one and year-two would be comparable. This study aims to provide information on the current lack of evidence on how students in Ethiopian medical schools with hybrid curriculum experience SDL. In addition, it contributes information for evidence-based decision of policymakers; it provides lesson for existing medical schools elsewhere to consider SDL; and it is a great input for future studies.

In the current study, several questions related to SDL are addressed: What do preclinical students who already have a bachelor degree understand by the term SDL? How do preclinical students from year-one reflect their level of SDL skill and does this differ from students in year-two? How do preclinical students from year-one perceive the support from the various elements of the hybrid curriculum on their SDL skills? Is this different from students in year-two?

## Methods

### Setting

The study was conducted at Aksum University medical school, Ethiopia. The medical school is among the 13 new medical schools in the country which adopted NIMC with the support of the Ethiopian Federal Ministries of Health and Education to tackle the significant shortage of doctors and burden of diseases in the country [34]. The medical curriculum is a five-year programme and designed for candidates aged below 35 years who already have a BSc degree in biomedical sciences. The first 2 years of the curriculum are preclinical years and the remaining 3 years are clerkship years [34]. It is a hybrid curriculum, some elements, such as weekly tutorial sessions are according to the PBL strategies, still, other elements, high number of lectures, stick more to traditional teacher-centred approach [34]. Case analyses in PBL tutorials are embedded in the first 2 years of the preclinical programme and students have to analyse multilevel PBL cases in a seven-step approach through tutorial one (pre-discussion), self-study, and tutorial two (reporting session) [12]. Multilevel PBL paper patient cases, which are constructed to address the core objectives of the preclinical years, are used to stimulate basic science learning. In the pre-discussion session (2 h long) students follow the first five steps of the seven-step approach and end-up with designing learning goals. Between tutorials is time allotted for students’ self-study to achieve all learning issues and get prepared for tutorial-two discussion.

### Participants

The study population was all year-one and year-two students of the medical school in Aksum. Almost all first-year (*n* = 30 from 31) and second-year (*n* = 32 from 38) medical students of Aksum University participated in the study. In both years students follow comparable learning activities, a combination of PBL based learning activities, like each one tutorial with problem analyses and traditional based learning activities, like a weekly high number of teacher-centred lectures. In both year-1 and year-2, there are no training of other activities explicitly focusing on SDL. Data were collected after students completed all their course work, at the end of year-1 and year-2.

### Design

We did a cross-sectional study [35] on first- and second-year medical students at the School of Medicine. A mixed-methods research design [36] was employed to explore students’ experience about their SDL skill and support of various elements of the curriculum on their SDL skill from March 26 – May 03/2019.

### Data collection

The validated Likert-based self-rate scale for SDL (SRSSDL) questionnaire developed by Williamson [37] was used to measure students’ perceptions of their SDL level. The questionnaire consists of 60 items related to students’ level of self-directedness in learning and they are arranged in five domains: *self-awareness*, *learning strategies*, *learning activities*, *self-evaluation*, and *interpersonal skills*. The scale was found reliable with Cronbach’s alpha coefficient for each domain varied between 0.71 and 0.79 [37]. Besides, the questionnaire designed by Dolmans & Schmidt [14] was used to explore students’ views about the influence of components of the hybrid curriculum on their SDL. The items are 20 in number and set in six themes: *discussion in the tutorial group*, *content tested*, *course objectives*, *lectures*, *tutor*, and *references*. The coefficient alpha for each theme was found between 0.51 and 0.82 [14]. All items were rated by students whether they behave always (5), often (4), sometimes (3), seldom (2), or never at all (1). A pilot study was conducted on four third-year medical students.

Two focus group discussions (FGDs), one containing eight participants from year-one and one containing year-two students were conducted using similar interview questions after the questionnaire survey was performed. The purpose of the FGDs was to get more insight and in-depth information about students’ understanding of SDL and the influence of curricular components on their SDL. The focus group was designed following the guidelines for Stalmeijer & colleagues [38] and its duration was ranged from 1:30–2:00 h long. Both FGD discussions were conducted in local language (Amharic) by two interviewers.

### Analysis

Quantitative data were analyzed using IBM SPSS version 20 (IBM, Armonk, New York, USA). Mean and standard deviation of students’ SDL scores were computed. Independent *t*-tests/ANOVA were used to investigate whether means of the scores differed between years, ages, marital status, and academic performance (cumulative grade point average, CGPA, of the particular year) of students. Students’ level of self-directedness in learning was ranked as low SDL (if they score within 60–140 range), medium SDL (141–220 score), or high SDL (221–300 score) [37]. We analyzed all results using α = 0.05. Moreover, we used Pearson correlation to evaluate whether and how strongly curricular components and SDL skills were related.

The focus group discussions were audiotaped and transcribed. The discussion contents were translated to English language by one of the focus group interviewers and checked by another staff with good English language skill. Subsequently, it was reviewed, coded, and then thematically analyzed by the main author and the two interviewers of the focus groups. There were repeated discussions among coders until agreement was reached.

### Ethical approval

Ethical approval was obtained from the Institutional Review Board (IRB) of Aksum University College of Health Sciences. Also, study participants were asked to participate in the study voluntarily and written informed consent was obtained by all study participants.

## Results

### Quantitative results

#### Overview of the personal aspects of participants

The response rate was 97% (30/31; 26 male, 4 female) for year-one and 84% (32/38; 28 male, 4 female) for year-two students. The study population in year-1 and year-2 were comparable (Table 1).

**Table 1:** Characteristics of year-1 & year-2 respondents, Aksum University, School of medicine, 2019

Demographic factors	Year-1	Year-2
	*N* = 30	*N* = 32
*Age*		
20–24	2	–
25–29	28	27
30–34	–	5
*Marital status*		
Single	20	19
Married	10	13
*Recent background degree*		
BSc	30	32
*Background education*		
None health	2	2
Medical Laboratory	1	1
Midwifery	6	4
Nursing	5	13
Optometry	1	1
Public Health officer	15	10
Psychiatry	0	1
*Academic performance (CGPA)*		
> 3.5	9	11
3.0–3.5	18	17
< 3.0	3	4

#### Students’ SDL ability

**Fig. 1:**
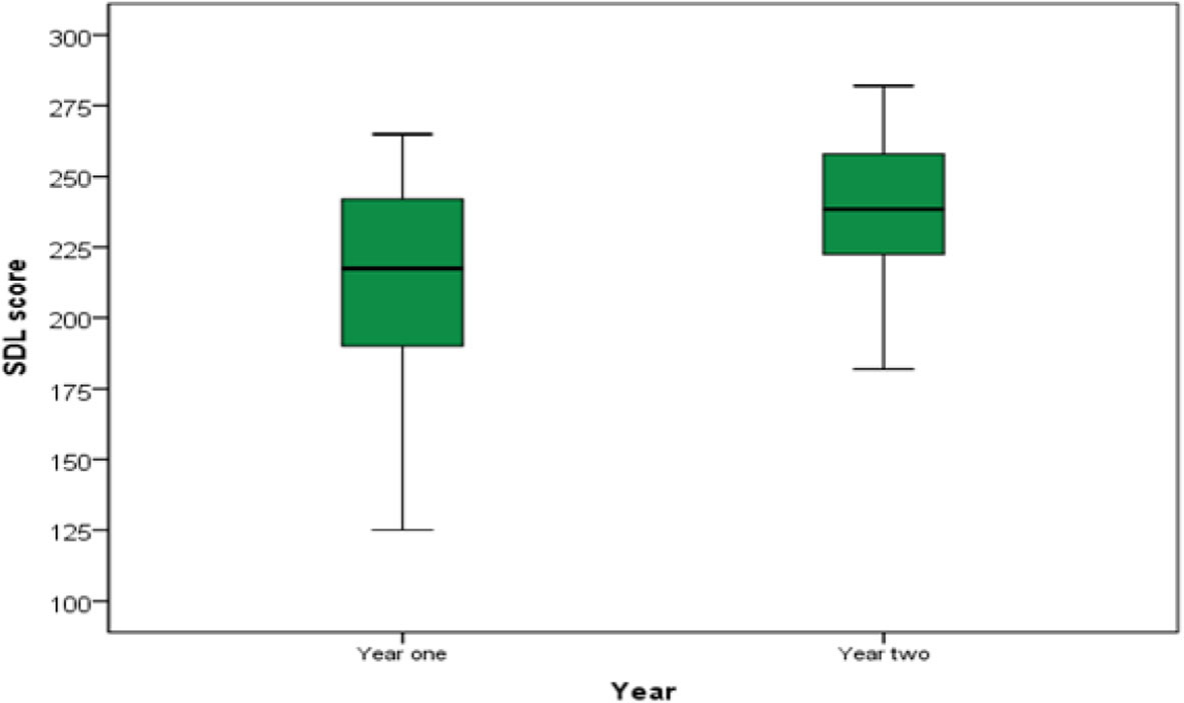
Box plot demonstrating the mean SDL readiness score of year-1 & year-2 students

The SDL readiness scale (SDLRS) questionnaire of year-1 and year-2 students was analyzed and compared. The overall mean SDL readiness score for preclinical students at Aksum University medical school was 225.63 (±34.11). Year-1 students had a mean SDL score of 212.3 (medium level) whereas year-2 students had a mean score of 238.2 (high level) (Fig. 1). A significant increase in SDL score on comparing students at year-1 with students at year-2 (*p = 0.002*) was found in this study.

In both year-1 and year-2, there were no significant effects of age, marital status, and academic performance on students’ SDL scores (Table 2).

**Table 2:** A comparison of the SDL Scores of medical students within the year of study based on age, academic performance, & marital status, Aksum University, School of medicine, 2019

Variables	Year-1		Year-2	
	Mean score (SD)	*P value*	Mean score (SD)	*P value*
Academic year	212.3 (36.26)	–	238.2 (26.90)	–
Age				
20–24	248.0 (24.04)	0.152	–	0.997
25–29	209.7 (35.91)		238.2 (28.18)	
30–34	–		238.2 (21.23)	
Academic performance				
> 3.5	213.3 (37.87)	0.934	252.5 (19.86)	0.066
3.0–3.5	213.0 (36.59)		228.6 (28.99)	
< 3.0	204.6 (43.02)		239.2 (20.17)	
Marital status				
Single	213.1 (39.96)	0.862	236.3 (28.53)	0.648
Married	210.6 (29.34)		240.8 (25.22)	

Interpretation of scores: Low (60–140); medium (141–220); high (221–300)

#### Influence of curricular components on students’ SDL skills

The perceived influence of tutorial discussion, content tested, module/case objectives, lectures, tutors, and dependence on suggested references/sources on the individual study were scored by both year-1 and year-2 students. Both year-1 and 2 students rated PBL tutorial discussion and tutors had a high influence on their independent learning; whereas, other curricular components such as lectures and testes had a low influence on their SDL ability (Table 3).

**Table 3:** Students’ perceptions of the influence of the New Innovative medical Curriculum (NIMC) components on their SDL skills, Aksum University, School of medicine, 2019

Class year of students	Curriculum components					
	PBL tutorial discussion	Content tested	Module objectives	Lectures	Tutors	Reliance on suggested references
Year-1 Mean (SD)	3.5 (1.13)	1.95 (0.52)	2.9 (1.03)	1.9 (0.6)	3.7 (1.04)	1.78 (0.6)
Year-2 Mean (SD)	4.2 (0.77)	2.1 (0.74)	3.8 (0.8)	2.0 (0.8)	4.1 (0.9)	3.2 (0.9)

*NB*: Ratings were from 1 (minimum) to 5 (maximum)

Correlation between curricular components and students’ self-rated SDL scores were also executed. Tutorial discussion and module/case objectives showed a strong correlation with SDL scores. Regarding the relationship between curricular components, the tutorial discussion was correlated strongly with both tutors and module objectives. Content tested was highly correlated with lectures (Table 4).

**Table 4:** Correlations among SDL scores and curricular components such as tutorial discussion, test, module objectives, lectures, tutors, and suggested references/sources, Aksum University, School of medicine, 2019

Variables	PBL tutorial discussion	Content tested	Module objectives	Lectures	Tutors	References/sources
SDL skills	.718a	.120	.648a	.085	.475a	.420a
PBL tutorial- discussion	–	.293^b^	.574a	.147	.599a	.366a
Content- tested	.293^b^	–	.180	.595a	.328a	.076
Module- objectives	.574a	.180	–	.070	.412a	.450a
Lectures	.147	.595a	.070	–	.142	.044
Tutors	.599a	.328a	.412a	.142	–	.178
References/sources	.366a	.076	.450a	.044	.178	–

^a^Correlation is significant at the 0.01 level. ^b^Correlation is significant at the 0.05 level

### Qualitative results

The focus group discussion from year-1 and the focus group discussion from year-2 provided in-depth information about students’ understanding of SDL and curricular components that support or hinder SDL. The findings are presented below, clustered in three themes: students’ definition for SDL; support of the teacher of SDL; and curricular components and activities that influence students’ SDL. Illustrative quotes are presented in italics.

#### Students’ definition for SDL

Students were asked to share their definitions of SDL and it could be understood that both cohorts have a certain level of understanding about SDL. Compared to year-1, Year-2 students showed a deeper understanding of SDL. Year-2 students identified different aspects of SDL in their definitions. In their definition, the concept SDL was described as the ability to identify learning needs, to distinguish relevant references to meet the identified learning issues, to use appropriate learning strategies in achieving the identified learning gaps, and evaluating the effectiveness of the learning outcomes. The following quotation shows the general view on how year-1 students defined SDL:

*‘….. I think now SDL means to me digging out and expanding the small direction we got in the class.’ [Year-1 student]*

#### Support of the teacher of SDL

The importance of support from teachers, particularly during year-one, is indicated in both focus groups as they played a vital role in making them self-directed learners.

*‘I think support from teachers is vital, especially at the beginning. We may be poor in identifying our gaps, may wish to read everything which is a problem for our survival, and may face difficulty how to get references….. For instance, I myself although completing year-1, I have still some problems how to use the internet and get reliable references.’ [Year-1 student]*

#### Curricular components and activities that influence students’ SDL

Students in both cohorts mentioned several factors that facilitate or deter effective individual learning. Both year-1 and 2 students indicated that, although they perceived they didn’t have experience of SDL in their background education, the hybrid curriculum exposes them for individual learning and promotes their SDL ability as the tutorial session is incorporated within it. Students mentioned that their SDL is highly motivated by tutorial discussion and tutors because the discussion on cases helps them to identify learning gaps and as a consequence, they become inspired to read more on that. The participants expressed it as:

*‘As you guess, we didn’t use SDL in our previous education. We used to expect everything from our teachers. SDL skill, as my colleagues said, needs background experience. I believe PBL tutorial discussion could break this barrier and made a great contribution to our current SDL ability. The tutorial discussion helped me to identify my learning needs and encouraged me to undergo individual study.’ [Year-1 student]*

*‘I should say the guidance, support, and feedback provided by tutors inspired my SDL …. they frequently asked students to clarify things which were very helpful to recognize what we didn’t clearly know.’[Year-2 student]*

Both year-1 and year-2 students associate SDL largely with PBL which is illustrated by the following quotations of a year-1 student followed by the quote of a year-2 student.

*‘Actually, I started practising SDL since I joined this medical school which incorporates PBL. I didn’t experience it in my background degree….’ [Year-1 student]*

*‘SDL is just having the motivation of teaching yourself on your own. It is exactly what we do in PBL….. you have learning issues which need further reading, so, you have to adjust your time and then select references to read …...I usually check myself whether I cover at least the most important learning issues because I want to actively participate in the tutorial discussion.’[Year-2 student]*

Participants from year-1 and year-2 expressed the positive relation between SDL and PBL because, in the PBL session, they felt challenged and supported to feel responsible for own learning. In the same way, during PBL session they gradually built confidence to reflect their knowledge and understanding to colleagues in tutorials, classes, and elsewhere.

On the other hand, the influence of module objectives on students’ SDL ability felt differently by year-1 and year-2 cohorts. Students in year-2 perceived module objectives as a key element of the new innovative curriculum that supports individual learning because of its integrated nature in all modules. The explanations why this happened were indicated in the focus group discussions. Students from year-1 and 2 stated their opinions:

*‘The module objectives have a positive influence on our SDL ability. I think this is due to the integration of basic sciences (multidisciplinary nature) in every module. This encouraged us to fully involved ourselves in connecting different points related to anatomy, physiology, microbiology, pathology, and pharmacology.’ [Year-2 students]*

*‘…..The introduction module which accounts for 16 weeks out of 45 weeks allocated for year-1 courses does not include PBL and mostly it is lecture-based which downgrade interactive sessions. I should say (because of this module) not that much’ [Year-1 students]*

Both year-1 and 2 cohorts similarly believed that curricular activities such as lectures seemed not motivating factors for self-directedness. It becomes clear that this curricular activity didn’t foster students’ SDL. Students perceived the curriculum is still dominated by lectures and it is not free from teacher-centred culture. Lectures were among the key factors that deterred SDL as indicated in the following statements:

*‘….. unfortunately, in my opinion, the main obstacle for our SDL was the lecture. Teachers tried to cover everything in non-interactive sessions and overwhelmed us with so many powerpoint slides, up to 2000, in a single course of a single system. Taking the tight schedule into account, reading all these notes didn’t give us time to go through reference books.’ [Year-1 students]*

*‘…………The lectures were poor in promoting us to identify our knowledge gaps.’[Year-2 students]*

*‘I think dumping a large number of lectures does not mean it is totally related to effective teaching-learning. Lectures have to be interactive, go with our level, cope-up with available time, and trigger SDL.’[Year-1 students]*

Moreover, both year-1 and 2 focus groups felt that the assessment which was mainly lecture-based does not stimulate their SDL. Students believed lecture-based exams deterred their SDL.

*‘We were assessed mainly from lecture contents. To score high marks you had to be assessment-oriented and just reading your lecture notes and memorizing what you read.’[Year-2 students]*

*‘To be honest, our exams were disrupting even for our PBL self-study.’ [Year-1 students]*

There was a similar perception of the impact of the country’s higher education culture on SDL. Students indicated that their teachers are products of conventional curricula and the majority of them have high power in deciding what and how they learn.

*‘Teachers…..I didn’t see them to motivate us for individual learning. They rush to cover their bulky and tiresome lectures in the class. In this case…..I found teachers better when they facilitate tutorial than lecturing in the class.’ [Year-1 students]*

*‘Some of us are still with the mindset of high dependency on teachers since we thought teachers know everything about the course.’ [Year-1 students]*

*‘I would say SDL had better be practised in other educational programs. Students joining the medical program could easily become popular with the method from the beginning.’ [Year-1 students]*

*‘Almost all of our teachers didn’t have this (SDL) experience in their background education. I suggest all teachers should be involved in training like what has been done for tutors.’[Year-2 students]*

## Discussion

This study aims to explore self-directed learning (SDL) ability of preclinical students and to assess how various elements of a hybrid curriculum influence students’ SDL. Year-1 and year-2 students are compared.

The overall students’ average SDL score was 225.63 which indicate high self-direction. In our study, first- and second-year students self-rated their SDL skill, 212.3 (medium level) and 238.2 (higher level), respectively. Significant differences were observed in SDL scores on comparing students at year-1 with students at year-2. This implied students’ SDL ability differed with a year of study. Our findings are in line with the study that revealed students from PBL curriculum to be progressively better self-directed learners across curricular years [17]. Nevertheless, our findings differ from a previous cross-sectional study on a hybrid curriculum at Dalhousie University Faculty of Medicine, Canada that shows no differences in students’ SDL scores between year-1 and 2 [19]. Our results are also different from cross-sectional studies on hybrid curricula in University of Toronto Faculty of Medicine and Indonesian medical schools where first-year medical students had significantly higher scores than second-year students [25, 39]. Besides, other longitudinal studies that followed undergraduate students’ SDL readiness in PBL hybrid and traditional medical curricula showed a significant drop in SDL readiness scores with medical training [28, 40]. The observed SDL differences between years in the present study might be due to the progressive positive effect of the NIMC, particularly PBL tutorials, on students’ SDL skills. Findings from focus group discussions show that SDL, particularly SDL in PBL, supports students to feel responsible for their learning. Lack of prior SDL experience in students’ background education might be a reason for first-year students to perceive a relatively lower level of SDL than those in year-2. Compared to year-1, students from year-2 provided the main elements of the SDL concept in their definitions showing some differences in practising SDL.

The findings of this study showed no significant effect of age on students’ SDL scores. This is similar to different studies elsewhere [25, 27]. On the contrary, it disagrees with other studies reported older students had significantly higher scores than younger students [28, 41]. Our finding probably implies students enrolled in the NIMC program have comparable age as all are within the adult population with a background degree. In adult learning theory older students are supposed to be with an equivalent ability of planning, conducting, and evaluating own learning [42].

It was noticed that there is no significant effect of academic performance (GPA) on students’ SDL scores. It is supported with the previous studies [21, 28, 43, 44] and disagreed with several studies reporting that SDL was found to be related to students’ academic performance [22, 24, 26]. A possible explanation for our finding might be the lecture-oriented test practised with great emphasis on fact learning and no room to assess the level of insight and reasoning as discussed within the tutorials in the medical school couldn’t promote students’ self-directed learning. According to Shah et al. [45], medical students may sometimes show a tendency to exercise a surface learning approach.

Regarding the influence of curricular elements on students’ SDL scores, PBL tutorial discussion and tutors were self-rated by students for their high influence on independent learning. Because of this, students generally perceived the hybrid curriculum promoting their SDL. The finding is in line with the report of Lee et al. [19] that stated a hybrid curriculum positively influence students’ SDL skills. However, our result is different from those of Harvey et al. [39] who reported the effect of a hybrid curriculum at the Faculty of Medicine, University of Toronto, Canada, didn’t foster students’ SDL. From the findings of qualitative data, both year-1 and 2 students indicated, although they perceived they didn’t experience SDL in their background education, the New Innovative Medical Curriculum exposed them for autonomous learning and promotes their SDL ability as the preclinical curriculum contains PBL within it. Students’ SDL was highly motivated by tutorial discussion and tutors. The discussion on cases provides students with many clues and helped them to identify learning gaps for independent learning [46]. Our study shows other curricular elements such as lectures and testes that had low influence on students’ SDL ability. This was supported with findings from focus group discussions which revealed these curricular activities that were not perceived by students as motivating factors for self-directedness. Students felt that the teaching-learning culture was still dominated by lectures and lecture-oriented tests. The teaching-learning system was not free from teacher-centred culture since the majority of teachers still had high power in deciding the learning process. Our findings disagreed with the study in Canada by Lee et al. [19] who reported students’ SDL ability was highly influenced by lectures. This difference might be due to the variation in the learning environment and lecture skills of faculty in both settings. A large body of literature suggests that teachers have to practice facilitation role more than the transmission of information [47, 48]. This reminds us of the importance of having teachers equipped with skills essential to implement SDL promoting curriculum [49].

Regarding the correlation among the curricular components and students’ SDL scores, tutorial discussion and module objectives showed a strong correlation with SDL scores. These quantitative findings suggested that both discussions in the tutorial group and module objectives powerfully related to students’ self-directedness. This finding would appear to differ with a study reporting that tutorial discussion and unit/case objectives showed a weak correlation with students SDL ability [19]. In the focus group interview, the influence of module objectives on students’ SDL ability felt differently by year-1 and year-2 cohorts. Students in year-2 identified module objectives as a key element of the curriculum that supports individual learning because of its multidisciplinary and integrated nature in all year-2 modules. Students in year-1 explained all modules of year-1 didn’t promote their SDL: for example, the ‘Introduction to Medicine’ module was perceived as less integrated and interactive. Furthermore, the tutorial discussion was strongly correlated with both tutors and module objectives. Our study noted that content tested was highly correlated with lectures. This finding implies that students tend to rely more on the content covered in lectures and tests [14].

This study has several limitations. It is limited because of a small sample size and lack of participants from teachers, although almost all preclinical students were encouraged to participate in our study. Its focus on a single Ethiopian medical school may limit the generalizability of the findings across different medical schools in the country and beyond. Another most important limitation is linked to the study design employed. The current study describes the students’ SDL ability at one time without following up their progression. Following students throughout the academic year or across years could better ascertain students’ progress in having SDL than a cross-sectional study. Moreover, because SDL ability was assessed by students themselves, it might limit the reliability of the findings. To address comprehensively, future research should consider the limitations observed in the current study to deeply investigate students’ SDL ability and benefits of hybrid curricular innovations.

## Conclusion

In the present study, first- and second-year medical students self-rated their SDL score as medium and higher level, respectively and significant differences are observed in comparing across years. The findings of this study show no significant effect of age and academic performance on students’ SDL scores. Some components of the hybrid curriculum are perceived as promoting SDL. PBL tutorial discussion and tutors show high influence on students’ independent learning; whereas, other curricular components such as lectures and tests show a low influence on students’ SDL. Tutorial discussion and module objectives show a strong correlation with student’s SDL ability. Tutorial discussion is correlated strongly with both tutors and module objectives. Content tested is highly correlated with lectures and this implies students tend to rely more on the content covered in lectures and tests which does not influence on SDL. Findings from qualitative data demonstrate that certain curricular components played a role in promoting students’ SDL. Tutorials analyzing problems played a major role in students’ self-directed learning abilities. Taken as a whole, the present study shows the New Innovative Medical Curriculum (NIMC) is still not free from teacher-centred culture since the majority of teachers still have high power in deciding the learning process and since all modules are still not well integrated and interactive. As most of the Ethiopian teachers are products of conventional curricula, continuous training may play a supportive role in making them skillful how to implement SDL promoting curriculum and how to promote students SDL. Most importantly, a further longitudinal study is needed to independently verify the actual level and behaviours of medical students’ SDL in a hybrid curriculum.

## Data Availability

The datasets used and/or analysed during the current study are available from the corresponding author on reasonable request.

## Abbreviations

CGPA: Cumulative grade point average
NIMC: New innovative medical curriculum
PBL: Problem-based learning
SDL: Self-directed learning
SDLRS: Self-directed learning readiness scale
SRSSDL: Self-rate scale for self-directed learning
